# Origami silicon optoelectronics for hemispherical electronic eye systems

**DOI:** 10.1038/s41467-017-01926-1

**Published:** 2017-11-24

**Authors:** Kan Zhang, Yei Hwan Jung, Solomon Mikael, Jung-Hun Seo, Munho Kim, Hongyi Mi, Han Zhou, Zhenyang Xia, Weidong Zhou, Shaoqin Gong, Zhenqiang Ma

**Affiliations:** 1 Department of Electrical and Computer Engineering, University of Wisconsin–Madison, Madison, WI 53706 USA; 2 Department of Electrical Engineering, University of Texas at Arlington, Arlington, TX 79019 USA; 3 Department of Biomedical Engineering and Wisconsin Institutes for Discovery, University of Wisconsin–Madison, Madison, WI 53706 USA

## Abstract

Digital image sensors in hemispherical geometries offer unique imaging advantages over their planar counterparts, such as wide field of view and low aberrations. Deforming miniature semiconductor-based sensors with high-spatial resolution into such format is challenging. Here we report a simple origami approach for fabricating single-crystalline silicon-based focal plane arrays and artificial compound eyes that have hemisphere-like structures. Convex isogonal polyhedral concepts allow certain combinations of polygons to fold into spherical formats. Using each polygon block as a sensor pixel, the silicon-based devices are shaped into maps of truncated icosahedron and fabricated on flexible sheets and further folded either into a concave or convex hemisphere. These two electronic eye prototypes represent simple and low-cost methods as well as flexible optimization parameters in terms of pixel density and design. Results demonstrated in this work combined with miniature size and simplicity of the design establish practical technology for integration with conventional electronic devices.

## Abstract (web summary)

Hemispherical format has been adopted in camera systems to better mimic human eyes, yet the current designs rely on complicated fabrications. Here, Zhang et al. show an origami-inspired approach that enables planar silicon-based photodetector arrays to reshape into concave or convex geometries.

## Introduction

Biological eyes are highly sophisticated and remarkably designed vision organs that have inspired biomimicry for several centuries. From lobster eye-inspired radiant heaters to moth eye-inspired anti-reflective coatings, human challenges have been solved by nature’s advice in a wide variety of applications^1, 2^. Also, the camera, which is undoubtedly the most revolutionary invention of mankind inspired by the eye, has immensely comforted, amused, and protected human lives. Today, technological advances have improved the quality of cameras with superior resolution, long focal lengths, and smart functionalities that are implemented in almost every consumer electronics system. In addition to these evolutions, reshaping conventional planar sensor systems into hemispherical formats would empower visual recordings with features that are beyond what state-of-the-art cameras can see, such as infinite depth of field, wider view angle, and lower aberrations^3^. Studying various eye systems in biology, most eyes have photoreceptors that capture and transduce photons into electrochemical signals laid either in concave or convex curvature. The concave array is mostly found in mammals as a camera or pin-hole type while the convex array is found in insects as a compound type. The camera or pin-hole eye has an outstanding quality of vision as it focuses light into an array of photoreceptors laid in a hemispherical concave structure (i.e., retina), allowing for clear identification of objects. The retina adopts the curvilinear shape that approximates the focal plane of the lens such that human eyes have large view fields and supreme focusing capabilities^4^. The compound eye has a wider-angle field of view via hundreds to thousands of ommatidia that are densely arrayed in a hemispherical convex structure for the sensitive detection of moving objects^5^. As such, biomimicry using semiconductor sensor systems structured in hemispherical formats would extend the capabilities of camera systems by utilizing the marvelous features of biological eyes.

Although numerous types of artificial eyes have been presented in the past, the inspired systems lacked the essential photo-detecting unit^6–9^. To take advantage of mimicking biological eyes in electronic imaging systems, photodetectors must essentially be represented in either hemispherical concave or convex formats rather than in the planar format typically found in conventional camera systems. Fabricating devices on non-planar surfaces, however, can be a major challenge because conventional fabrication techniques were developed for planar wafers or plate materials in the semiconductor industry. The simplest approach to deforming sensors was to mount optoelectronic components on a flexible printed-circuit board (FPCB), but this could only achieve hemicylindrical photodetector array designs^10^. Specialized techniques to apply stress on and bend ordinary planar silicon wafer-based CMOS image sensors have been introduced as well, which have been successful in inducing minor curvatures^11, 12^. The available techniques for direct fabrication on non-planar surfaces, such as soft lithography, mechanical molding, and lens-assisted lithography, are consequently complicated and expensive, and have very specific requirements. Encouraged by the promising prosperity in non-planar devices, novel strategies have been investigated to circumvent the limits set by non-planar surfaces while utilizing mature semiconductor fabrication techniques for economic consideration. For instance, transfer printing of ultrathin semiconductor nanomembranes onto rubber- or plastic-like substrates transformed the shape of high-performance electronics and optoelectronics into flexible and stretchable formats^13–18^. These unusual semiconductor devices on complex curvilinear surfaces are versatile in various areas due to their new degree of design freedom and biomimicry merits, including the hemispherical photodetector array^19–23^. Successful integration of stretchable photodetectors with camera systems has demonstrated concave and convex curvilinear photodetector arrays for the hemispherical electronic eye camera that mimicked the human eye^21^ and compound electronic eye camera that mimicked the arthropod eye^22^. In both designs, a large array of thin silicon photodiodes separated by serpentine traces of metal for electrical interconnects were originally fabricated on a planar host substrate and transfer-printed onto rubber substrates. Upon hydraulic actuation, the array on rubber deformed and stretched into either a concave or convex structure, where the geometry of the serpentine wire tortuosity deterministically transformed the layout without electrical or mechanical failure. Both concave and convex camera systems that used silicon optoelectronics were groundbreaking toward camera evolution, but the requirement of hydraulic actuators may be bulky in many miniaturized camera systems for consumer devices. Also, the large separation distance between photodetector pixels reserved for electrical traces that are in the micrometer range may pose limitations in resolution optimization. An approach that is both compatible with commercially available imaging systems and has flexible optimization parameters is desirable for such hemispherical photodetector arrays to become more practical.

Here, we present a unique origami-inspired approach, combined with semiconductor nanomembrane-based flexible electronics technology, to build dense, scalable, and compact hemispherical photodetector arrays. Originated as an art of paper folding, origami and kirigami were recently utilized to assemble three-dimensional structures with micro/nanomembrane materials to allow an increasingly wide range of applications^24–28^. Precut membranes have been structured into numerous types of interesting three-dimensional assemblies via buckling and folding to form various electronic components, including antennas, solar cells, batteries, nanogenerators, waveguides, photodetectors, and metamaterials^29–37^. Although a similar approach of forming curved silicon photodetector hemisphere was introduced in the past, optical imaging using the hemisphere array has not yet been demonstrated^38^. In this work, the folding mechanism is implemented for both concave and convex curvilinear photodetector arrays with single-crystalline silicon nanomembranes. The low flexural rigidity of single-crystalline nanomembrane allows high-performance photodetectors to bend with microscale radius of curvature^39^. Combining the origami-inspired approach with the transfer printing of advanced inorganic nanomembranes on flexible substrates, high-performance hemispherical electronic eye camera systems are fabricated to allow for unusual imaging that could not be done with conventional camera systems. Furthermore, the origami-based fabrication eliminates the use of metal wires in-between pixels for the connection of sparsely arrayed devices (as seen in other similar systems that limited resolution optimizations), as well as eliminating the need for the sophisticated actuators that were used to form the hemispheres^21, 22^.

## Results

### Geometric origami for hemisphere-like silicon optoelectronics

**Fig. 1:**
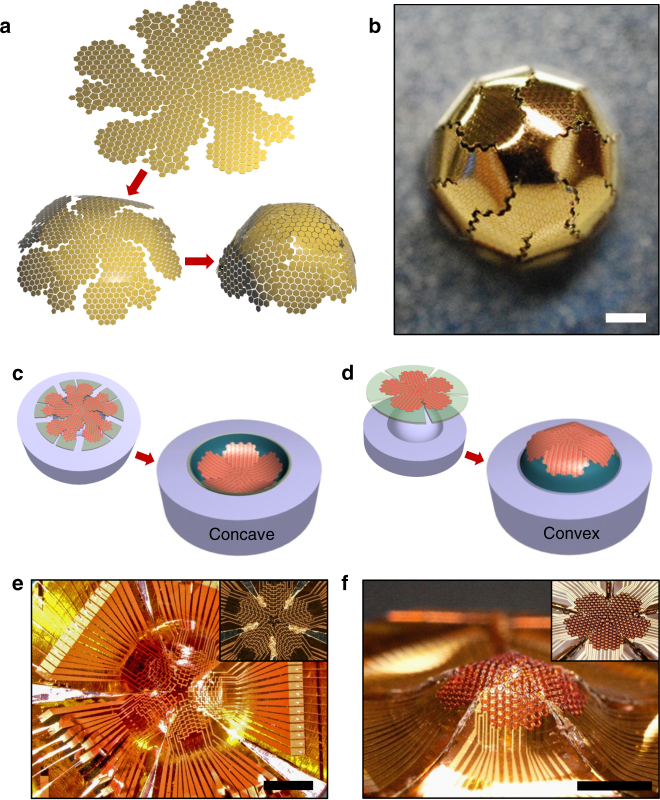
Geometric origami of silicon optoelectronics for the hemispherical electronic eye. **a** Schematic illustration of the net of half truncated icosahedron being folded into a hemisphere. 676 polygon blocks consisting of pentagons and hexagons were mapped into a net of subdivided half truncated icosahedron which was then folded to form a hemisphere. **b** A photograph of the half truncated icosahedron based on polygon blocks of metal-coated silicon nanomembranes printed on a flexible polyimide film. The completed net was folded into a convex hemisphere by inserting the net into a circular hole of a metal fixture. Scale bar, 1 mm. **c** Schematic illustration of the net of half truncated icosahedron based on silicon nanomembranes pressed into a hemispherical concave mold. **d** Schematic illustration of the net of half truncated icosahedron based on silicon nanomembranes covered on a hemispherical convex mold. **e** A photograph of a silicon optoelectronics-based hemispherical focal plane array formed using the concave mold-based origami approach shown in **c**. Inset image shows the flat focal plane array before folding. Scale bar, 2 mm. **f** A photograph of a silicon optoelectronics-based convex hemispherical eye camera formed using the convex mold-based origami approach shown in **d**. Inset image shows the flat eye camera before folding. Scale bar, 2 mm

Figure 1 illustrates the concept of geometric origami used for the photodetector array. In geometry mathematics, a quasi-spherical solid is formed using one of the renowned Archimedean solids—the truncated icosahedron—which is a combination of multiple pentagonal and hexagonal faces, typically found in soccer balls or buckminsterfullerene molecules. As presented in Supplementary Fig. 1a, a net of half truncated icosahedron was first mapped and cut on the flexible substrate, followed by folding the net to create a quasi-hemisphere. The edges of the hemisphere-like structure could be further smoothed out by dividing the large pentagonal and hexagonal faces into even smaller polygon faces as presented in Supplementary Fig. 1b. The subdivided icosahedron not only smoothed out the edges of the hemisphere, but also allowed more pixels inside the geometry to improve fidelity. As an example, Fig. 1a represents a schematic illustration where the 676 polygon blocks were mapped into a net of subdivided half truncated icosahedron which was then folded to form a hemisphere. Figure 1b shows a photograph of the folded truncated icosahedron using metallized silicon nanomembrane blocks, printed onto a flexible polyimide substrate. The entire microfabrication of electronic devices, including silicon etch, metal deposition, and device passivation, were completed in a planar format prior to deformation, simplifying the process flow of curvilinear semiconductor devices by leaving the deformation mounting to the last step and thus preserving the feasibility of most semiconductor fabrication techniques. As a result, a simple and practical method to integrate well-developed planar devices onto complex curvilinear surfaces was achieved, enabling diverse applications that were difficult to address using conventional means. As presented in Fig. 1c, d, the net may be folded upwards for a concave hemisphere or downwards for a convex hemisphere. For instance, the concave array may be used to mimic the retina in either pin-hole- or camera-type mammalian eyes while the convex array may be used to mimic the ommatidia in a compound eye. Using nanomembranes combined with flexible substrates, the ultrathin photodetector array further bends to yield a smooth hemisphere. Figure 1e, f represents the abovementioned concave and convex photodetector arrays, respectively, using the origami approach. They were then analyzed and are discussed in detail here in later sections.

### Si nanomembrane-based photodiodes for origami optoelectronics

**Fig. 2:**
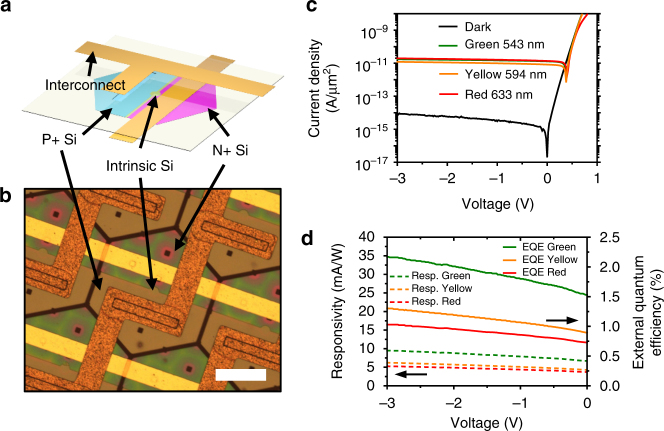
Electrical properties of a silicon optoelectronic device used for the electronic eyes. **a** Schematic illustration of a hexagon-shaped silicon nanomembrane-based photodiode used for the electronic eyes. An array of such photodiodes were printed and fabricated on a pre-cut flexible polyimide substrate. **b** Optical microscope image of the photodiodes. Scale bar, 50 μm. **c** Current density–voltage characteristics of the photodiode in the dark and under the illumination of lasers with wavelengths of 543 (green), 594 (yellow), and 633 nm (red). **d** Responsivity and external quantum efficiency of the photodiode under the illumination of lasers with green, yellow, and red wavelengths. The laser light intensities were 5 mW for the green and yellow wavelengths and 7 mW for the red wavelength. Green laser was used for the rest of this study

Silicon-based lateral P–i–N photodiodes were used as sensors in this study due to their broad spectrum, as well as the large bandgap of silicon and the fast response of the P–i–N structure. The schematic illustration shown in Fig. 2a and the optical microscope image in Fig. 2b represent the photodetector unit implemented in the electronic eyes. The photosensitivity results of the photodetector diode are shown in Fig. 2c. The measured dark current density was lower than 1 × 10^−14^ A μm^−2^ up to a –5 V bias and weakly dependent on the reverse-bias voltage. The photocurrents measured at –3 V under the illumination of three visible lasers, including green (543 nm), yellow (594 nm), and red (633 nm) lasers were 1.74 × 10^−11^ A μm^−2^, 1.21 × 10^−11^ A μm^−2^, and 1.95 × 10^−11^ A μm^−2^, respectively. The measured current densities of the photodetectors at the different power levels exposed with each visible laser are shown in Supplementary Fig. 2a–c. The ratio between the photocurrent and the dark current showed about a 10^4^ fold difference. The calculated photo responsivity was 9.49 mA W^−1^, 6.24 mA W^−1^, and 5.26 mA W^−1^ at –3 V under the green, yellow, and red lasers, respectively, as shown in Fig. 2d. The external quantum efficiency (EQE) was calculated using the photocurrent and the incident light power. At –3 V, the EQE was 2.2%, 1.3% and 1.0% for the green, yellow, and red lasers, respectively. Imaging with these photodetectors was performed using the green laser, as the photodiodes were most responsive to green light.

### Origami optoelectronics for hemispherical focal plane array

**Fig. 3:**
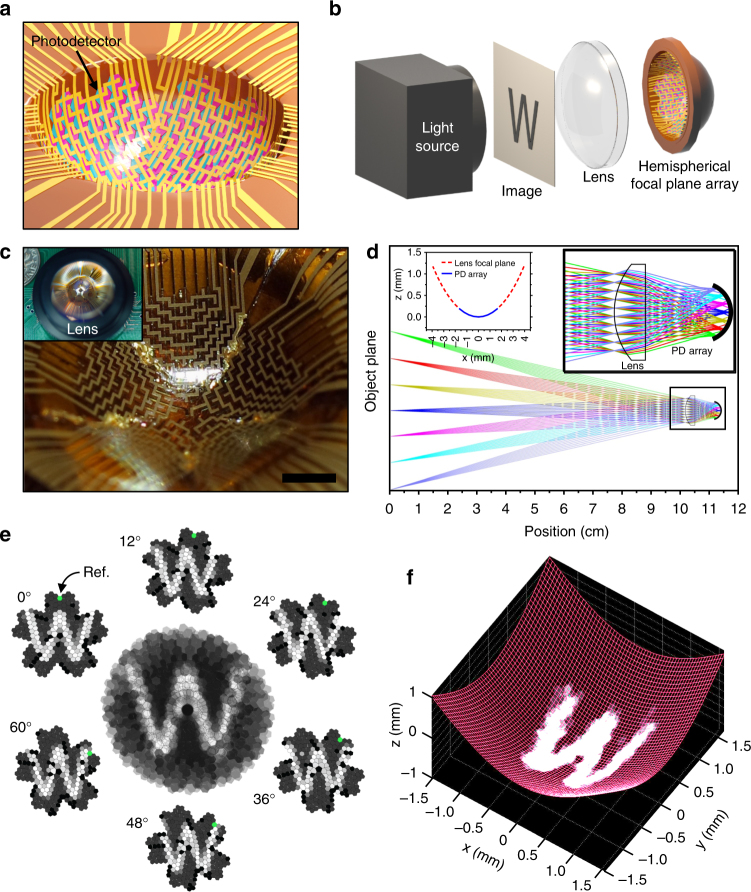
A concave hemispherical electronic eye camera system using origami silicon optoelectronics. **a** Schematic illustration of the hemispherical focal plane array (FPA) based on origami silicon optoelectronics. Fully formed photodiode array fabricated into a net of half truncated icosahedron was pressed and folded into a concave mold to create the hemispherical geometry. **b** Optics setup of the hemispherical electronic eye system shown using a schematic illustration with a light source, imaged object, and plano-convex lens to the left of the FPA. **c** A photograph of the hemispherical FPA based on origami silicon optoelectronics. Inset image shows a photograph of the electronic eye system with the plano-convex lens integrated on top of the FPA. Scale bar, 1 mm. **d** Ray patterns traced from different angles plotted against the position from the object plane. Right inset plot shows a magnified view of the dotted box shown in the plot. Left inset shows the calculated focal plane of the ray passing through the plano-convex lens (dotted red curve) and measured focal plane of the silicon optoelectronics array (blue curve). **e** High-resolution image of the letter ‘W’ acquired from the hemispherical electronic eye camera. The image was scanned from 0° to 60° in 12° increments for the refined imaging. Each inset image shows a snapshot at each degree angle, with the reference photodiode highlighted in green. **f** High-resolution image of the letter ‘W’ acquired from the hemispherical electronic eye camera matching the concave hemispherical surface of the FPA

Figure 3a presents a schematic illustration of the hemispherical focal plane array (FPA) based on origami silicon optoelectronics. The array was designed such that the pixels were laid out to form a large net of subdivided half truncated icosahedron, where each pixel contained a single photodetector and was shaped into either a pentagon or a hexagon, and electrically connected by metal interconnects. A macroscopic view of the hemisphere formed with this combination of polygons is represented with paper origami, as presented in Supplementary Fig. 3. In total, there were 281 photodetectors in the hemispherical array, and each was adjacent to one another. The inner diameter of a single hexagonal photodetector was 113 μm, as presented in Supplementary Fig. 2d. The net was first fabricated on a planar format where conventional optoelectronics processes involving high temperatures and chemical solvents were utilized and transfer-printed onto a flexible polyimide substrate. Once the fabrication and passivation of the device was complete, the flexible net of half truncated icosahedron was mounted onto a concave fixture to mechanically transform it into a hemisphere. To precisely mount and fold the net onto a fixture, a metal-based hemisphere (concave) fixture and a polydimethylsiloxane (PDMS)-based reverse (convex)-hemisphere pressing mold were prepared. The net was centered onto the reverse mold, where the adhesion of the PDMS-based reverse mold temporarily held the net during the mounting process. The mounting process was completed by coating the metal concave fixture with a thin layer of epoxy glue and gently pressing the reverse mold with device onto the fixture. This process flow is applicable to curvilinear surfaces with different curvatures and was verified by mounting the net onto two concave fixtures with different radii (*r* = 2.27 mm and 7.20 mm) of curvature of the hemisphere, as presented in Supplementary Fig. 4. Supplementary Fig. 4a and b describes the detailed design parameters of the metal concave fixtures with small and large radii, respectively. During the mounting process, mechanical deformation was introduced to each pixel. However, the low flexural rigidity from the extremely small thickness of the silicon nanomembrane used as the photodetector material allowed the mechanical deformation to have a negligible impact on the performance of the device^40^. The performance of the photodetector started to degrade when the radius of curvature reached 1.5 mm, as presented in Supplementary Fig. 5. The layer thicknesses of the silicon nanomembrane, metal interconnects, and polymer passivations can be reduced to achieve curvilinear photodetector arrays with smaller radius of curvature. For instance, a 20 nm thick silicon nanomembrane photodetector could wrap around the cladding layer of a single mode fiber to detect light leakage, which typically has a diameter of 125 μm^39^. It is important to carefully control the mechanical neutral plane of the device, such that minimal stress is applied on the most fragile part of the device^13^.

A simple camera system using a hemispherical FPA was assembled as presented in Fig. 3b. A plano-convex lens (10 mm diameter and 10 mm focal length) that focused light was placed in-between the array and the image. Figure 3c shows a photographic image of the FPA, with the inset image showing the assembled device including the plano-convex lens. Individual components, including the laser, image, lens, photodetector array, etc., were placed on a rail and allowed for flexible adjustments to the imaging setup, as shown in Supplementary Fig. 6. Figure 3d represents the simulated focal plane of the camera system. The distance between the plano-convex lens and the focal plane array was approximated by simulating an object reflected on a planar focal plane behind the lens. As shown in Supplementary Fig. 7, the focal plane from the planar plane of the lens was best approximated in the range of 7.0–8.5 mm, which agreed well with the back focal length (8.18 mm) provided by the lens manufacturer. With proper adjustments to the lens position, the focal plane had acceptable detection accuracy with the photodiode array, as shown in the left inset plot in Fig. 3d. Imaging of the object was also performed with the FPA mounted on a larger radius of curvature (7.20 mm) to demonstrate the origami photodetector array’s potential for hemispheres with various radii of curvatures. A plano-convex lens with a larger focal length (10 mm diameter and 20 mm focal length) was used for the photodiode array with the larger radius (7.20 mm). The focal plane for the photodiode array with the larger radius (7.20 mm) also had acceptable detection accuracy, as presented in Supplementary Fig. 8. In addition, the distance at which the camera system should be placed was calculated using the ray traces plotted against the distance between the object and the camera system. With the hemispherical focal plane’s radius of curvature fixed at 2.27 mm, the position of the plano-convex lens was calculated to be 10.3 cm away from the object plane (for a larger radius of 7.20 mm, the distance was increased to 20 cm).

Figure 3e, f shows the images obtained from the hemispherical FPA. Multiplexers allowed for the recording of signals from the large array of photodetectors in the matrix. The array of photodetectors with rows and columns of metal interconnects was connected to ten CMOS analog multiplexer circuits, where two 8-to-1 multiplexers controlled each side area of the half truncated icosahedron net. Imaging using the photodetector array was controlled with a computer programmed design platform in a passive matrix format. The recording mechanism and the multiplexer layouts, as well as the printed-circuit board (PCB) layout, are presented in Supplementary Fig. 9. The dense array of the photodetectors imaged the letter ‘W’ with relatively high-spatial resolution. To further improve the image quality and eliminate the dark spots from defective pixels, a sequence of images was collected while rotating the imaged object. Images were taken after rotating the image counterclockwise in 12° increments from 0° to 60°. Ideally, the camera system should be rotated, rather than the imaged object, as the object remains stationary when a photo is taken. Rotation of the camera system was limited in the setup as shown in Supplementary Fig. 6, thus the imaged object was rotated instead, mimicking the clockwise rotation of the camera system. A total of six images were combined and reconstructed to obtain a scanned, improved-quality image as shown in the middle image of Fig. 3e. Each of the six smaller images in Fig. 3e around the middle scanned image represents single scanning at a given rotation angle, with the reference photodiode shown in green for easier visualization of rotation. A high-resolution image of the letter ‘W’ acquired from the hemispherical electronic eye camera was rendered using numerical computing software to match the hemispherical surface of the FPA, as presented in Fig. 3f. It is expected that an image with higher resolution may be achieved by increasing the number of scans. The same set of experiments were performed for the photodetector array with the larger radius of curvature (7.20 mm). As presented in Supplementary Fig. 10, for the larger radius of curvature, a slightly deteriorated and blurred letter was detected as compared to the image from the smaller radius, possibly due to optical aberrations associated with an imperfect FPA and lens combination. It should also be noted that the design of truncated icosahedron was not optimized for such a large radius of curvature, which created blind spots within the FPA. Nevertheless, the use of hemispherical FPAs largely benefited from the simplified optical elements required to image an object, as recording with planar FPA requires complicated optical systems to eliminate off-axis aberrations such as astigmatism, field curvature, and coma. This not only saved cost, but also allowed for a much simpler and compact camera design.

### Origami optoelectronics for artificial compound eye

**Fig. 4:**
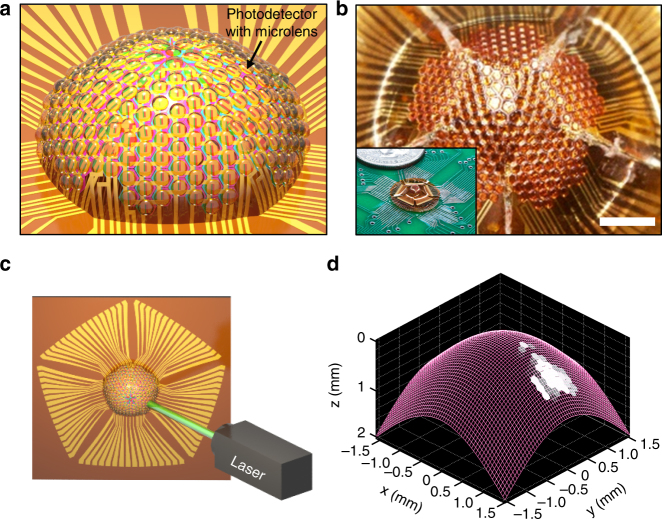
A convex hemispherical electronic eye camera system using origami silicon optoelectronics. **a** Schematic illustration of the convex hemispherical electronic eye camera based on origami silicon optoelectronics. Fully formed photodiode array fabricated into a net of half truncated icosahedron was covered and folded onto a convex mold to create the hemispherical geometry. **b** A photograph of the convex hemispherical electronic eye camera based on origami silicon optoelectronics. Each photodiode is integrated with a polymer microlens to mimic the corneal lens in a compound eye. Inset image shows a photograph of the compound electronic eye system mounted on a printed circuit board. Scale bar, 1 mm. **c** Optics setup of the compound electronic eye system shown using a schematic illustration, where the point laser is illuminated from an incident angle of 36°. **d** Image of the laser point acquired from the compound electronic eye camera matching the convex surface of the camera

Different from concave FPAs where light is focused onto with lens, a photodiode array may be folded in a reverse manner for potential compound eye mimicking cameras that do not need any external optics. The same fabrication technique can be used to create a convex array, with minor modifications to the individual photodiodes to include microlenses. Biological eyes with photoreceptors arranged in a convex format are typically found in compound eyes. The structure of compound eyes differs from mammalian eyes as they are comprised of ommatidia on the convex surface. In each ommatidium, a tiny corneal lens focuses incoming light rays onto a single photoreceptor inside of it. Such imaging elements can be artificially created on top of each photodetector using the simple photoresist reflow approach^41–43^. A microlens placed on top of each detecting unit maximizes the amount of light delivered to the photodiode by accepting light from large incident angles (Supplementary Fig. 11). A slight decrease in incident light was observed with the microlens at 0°, which was attributed to the light absorption in the photoresist, but the loss was negligible and the lens transmitted a higher incident light percentage at larger incident angles. Whereas an actual ommatidium consists of other elements (like pigment cells and crystalline cones, in addition to the corneal lens, that all together focus light and isolates itself from neighboring ommatidia), the convex array demonstrated in this report lacks such isolating elements. However, it shows the proof of concept that complex elements like the corneal lens can be fabricated on the photodetector array formed using the origami approach. Similar to the concave hemispherical FPA, the hemispherical electronic eye that was convex in shape was formed using the same net of subdivided half truncated icosahedron, with a radius of curvature of 2.27 mm. With the photoresist microlens fabricated on top of each photodetector, the net was mounted on a convex fixture as shown in the schematic illustration in Fig. 4a. The mounting process for convex array was completed by coating the convex fixture with a thin layer of epoxy glue and pressing down the net with a reverse mold. A photographic image of the device before being mounted on the convex fixture is presented in Supplementary Fig. 12. Figure 4b shows a photographic image of the convex hemispherical electronic eye and its inset image shows the device mounted on the PCB system. To demonstrate its ability to image with a wide field of view, a narrow laser beam was fired at an angle of 36° from the PCB plane, as illustrated in Fig. 4c. Figure 4d shows the image of the laser light acquired from the electronic eye camera matching the convex surface of the photodetector array, with brighter regions indicating the photodetectors of the convex electronic eye camera that detected the laser light. Although the convex design of the photodetector array enabled peripheral vision, the scanned image was closer to a blurry spot rather than a detailed single point. This was due to the large acceptance angle of the device that led to an overlapping of the light received by the adjacent diodes with microlenses. Additional biomimicry elements that isolated each photodiode and optimized acceptance versus inter-ommatidial angle could eliminate these adverse effects. As shown in this conceptual design, the convex hemispherical camera benefits from its capability to detect light from wide angles without any need of external optics like the camera-type eye. Such aspects are especially useful for visually controlled navigation and optometer responses that do not necessarily require extreme resolution, but require wide view angles and minimized device layouts. With further optimizations in the optical components by adding layers that mimic the pigment cells and crystalline cones, a compound electronic eye system that has panoramic vision may also be capable.

## Discussion

The biomimicry of eyes demonstrated in this report utilized the simple origami approach of deforming flexible electronics into hemispherical formats, which successfully generated two very important camera systems with a large number of photodetectors in a dense array. The density of the array can further be expanded by splitting the polygon blocks into smaller blocks or by attaching more pixels around the array. Moreover, the fabrication process can be made compatible with existing CMOS sensor technology with extremely high densities by releasing the array of CMOS sensors fabricated on silicon-on-insulator (SOI) wafers and origami-deforming the array at the last step. The conventional silicon manufacturing techniques used to fabricate the photodetectors, as well as the miniature size of the finished device, are beneficial to advance such an approach into commercial electronic systems. The easily scalable pixel density and the simplicity of the device structure are the key features of this method. Future research includes developing tunable hemispheres for the origami optoelectronics and mounting mechanisms for easy integration with other electronics. Furthermore, other convex isogonal polyhedral concepts, such as dodecahedron or rhombicosidodecahedron, may be employed as origamis to create unusual optoelectronics or electronics in hemispherical formats. Applying this concept to state-of-the-art digital cameras that capture high quality images or surveillance cameras using infrared night vision are also desirable which would further expand the capabilities of cameras.

## Methods

### Fabrication of Si-based photodiodes on flexible film

The fabrication of both concave and convex hemispherical eyes started from a lightly p-doped SOI wafer (SOITEC^TM^) which had a 270 nm device layer and 200 nm buried oxide (BOX) layer. The wafer was patterned and heavily doped with boron and phosphorus to form N+ and P+ regions using ion implantations (boron, dose of 4 × 10^15^ cm^−2^ and an energy of 20 KeV, and phosphorus, dose of 4 × 10^15^ cm^−2^ and an energy of 30 KeV), followed by diffusion at 950 °C for 20 min in a 5% O_2_, 95% N_2_ ambient atmosphere. An array of etch-holes was made using photolithography and reactive-ion etching (RIE) to partially expose the BOX layer, and the processed top Si nanomembrane layer was released by immersing it in concentrated hydrofluoric acid (49%) for 2 h. A flexible polyimide film (Kapton HN; Dupont; 127 μm) was prepared by laser cutting the film (A-laser) to match the half truncated icosahedron pattern of the array. The patterns of the photodiode array and flexible substrate both corresponded to a shape consisting of one pentagon surrounded by five hexagons, so that the finished array could be wrapped onto a hemispherical fixture. The Si nanomembrane was directly transferred onto the polyimide film by pressing the adhesive-coated (SU-8 2; Microchem; 2 μm) polyimide film against the released nanomembrane. During this process, a modified mask aligner (MJB-3; Karl Suss) was used to perfectly align and transfer the nanomembrane to the precut polyimide substrate. After curing the adhesive, the silicon nanomembrane was patterned and etched (RIE) into polygon blocks to isolate the pixels, and the SU-8 (SU-8 2; Microchem; 2 μm) passivation layer was patterned with via-holes, followed by the deposition of first metal interconnects (Ti/Au = 30/250 nm). Adding another SU-8 via-hole layer with second metal interconnects and the final SU-8 passivation layer concluded the device fabrication process. These detailed processes are described with schematic illustrations in Supplementary Fig. 13.

### Fabrication of polymer microlens for compound eye

For the convex hemispherical electronic eye, a microlens was fabricated on each SU-8 (4 μm) passivated photodiode for a wider view field. The fabrication involved photolithography of a thick photoresist (AZ4620; MicroChemicals; 40 μm) and thermal reflow. After isolating the photoresist with photolithography, oven heating for 15 min at 95 °C caused the photoresist to reflow to a near-convex microlens. This process is described with schematic illustrations in Supplementary Fig. 14.

### Origami process of Si optoelectronics

Mounting the photodetector arrays used the same procedures for both concave and convex arrays. Either the concave or convex fixture was first coated with an adhesive layer and the finished photodetector array (without a microlens for the concave array and with a microlens for the convex array) was carefully pressed against the fixture using a reverse PDMS mold. Finally, the device was mounted onto the PCB using gold wire bonding.

### Measurement and analysis

The measurements of the photodiode were performed using an HP 4155B Semiconductor Parameter Analyzer. Before the photodetector array was folded and mounted onto the hemispherical fixture, it was measured on a planar probe station with laser lights striking perpendicular to the device plane. Three different helium neon lasers emitting green (05-LGR-193; Melles Griot), yellow (25-LYR-173; Melles Griot), and red (1137 P; JDSU) lights were used for this study. The normalized current density was calculated for a single hexagonal photodetector for three laser beams. The concave camera system mounted on the lateral rail collected images from a beam expanded (15 × Complete Beam Expander; Edmunds Optics) green laser illuminated through a precut pattern of a letter ‘W’ and a plano-convex lens (#63–471; Edmunds Optics for small radius hemispherical FPA, and #63–473; Edmunds Optics for large radius hemispherical FPA) by recording photocurrents generated at each photodetector. This process was repeated with the imaged object (the letter ‘W’) rotated counterclockwise in 12° increments for six consecutive imaging steps. These were then combined to obtain scanning mode collection data and improve the effective resolution. The convex camera system mounted on the lateral rail recorded photocurrents generated at each photodetector from a narrow green laser illuminated from an angle of 36°.

### Data availability

The data supporting the findings of this study are included within the paper and its Supplementary Information, or available from the corresponding author upon reasonable request.

## Electronic supplementary material

Supplementary Information
