# Supplementary material for: Origami silicon optoelectronics for hemispherical electronic eye systems
Source: Nat Commun. 2017 Nov 24;8:1782. doi: 10.1038/s41467-017-01926-1 (PMC5701179; doi:10.1038/s41467-017-01926-1)
Supplement: Supplementary file 1 — Supplementary Information [file 41467_2017_1926_MOESM1_ESM.pdf]

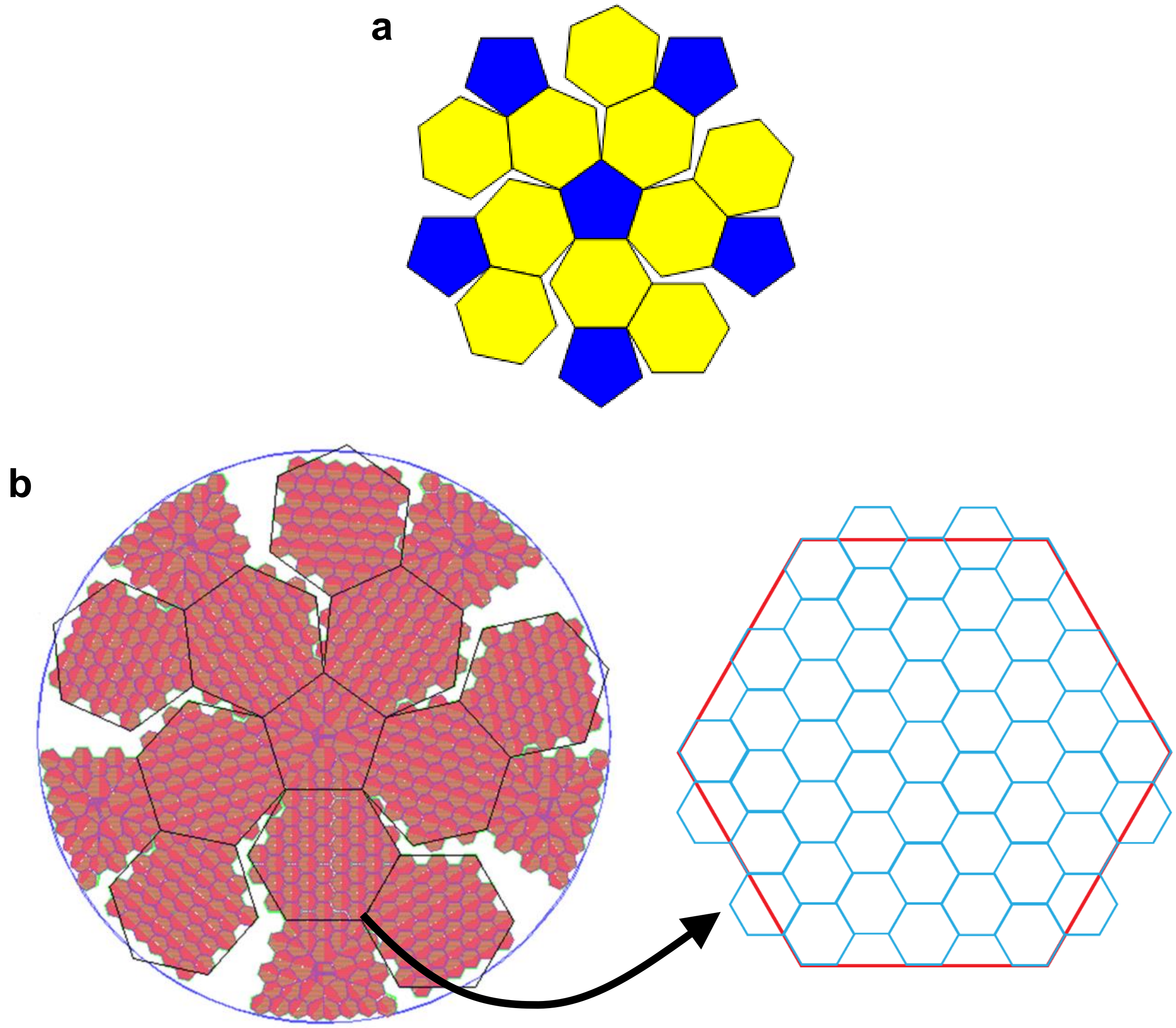

Supplementary Figure 1 | Geometric origami using truncated icosahedron. (a) A map of pentagon-centered half truncated icosahedron consisting of 10 hexagons and 6 pentagons that can be folded to form a quasi-hemisphere. (b) Geometry of subdivided half truncated icosahedron for producing a hemisphere-like structure. Each pentagon and hexagon can be a pixel (i.e., a single photodetector), and the subdivision can get even more detailed for higher pixel densities and a smoother hemisphere.

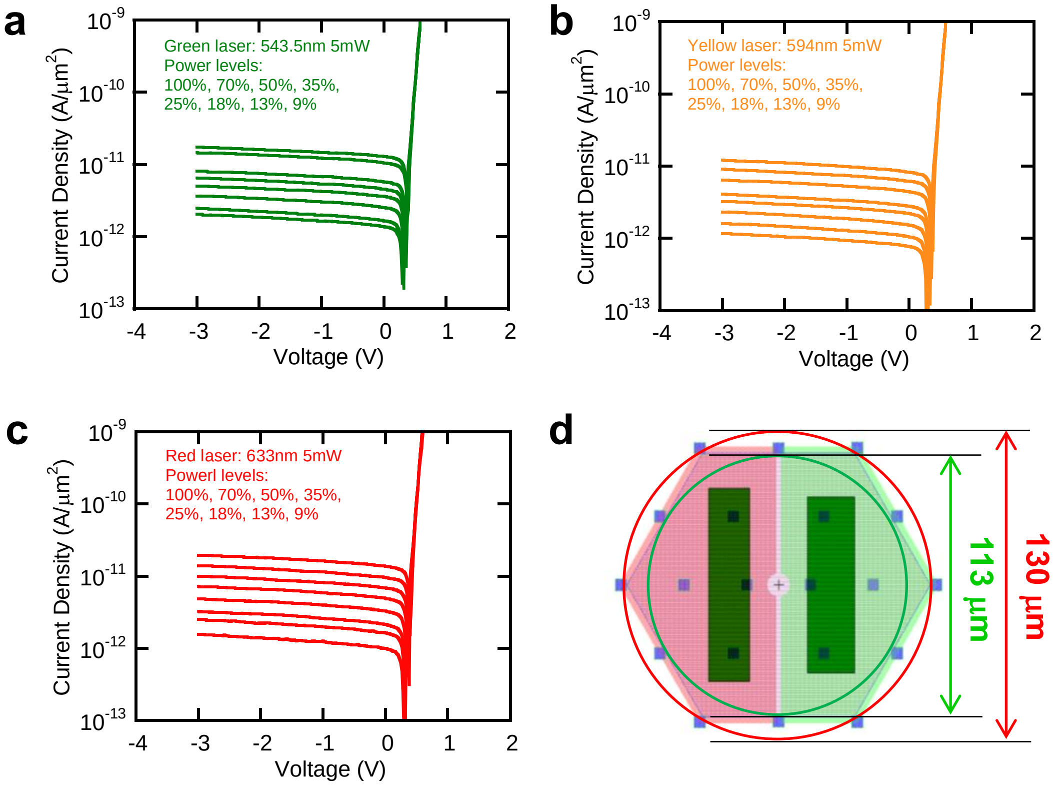

Supplementary Figure 2 | Detailed characteristics of a silicon photodetector. Current density–voltage characteristics of the photodiode under the illumination of lasers at various power levels with wavelengths of (a) 543 (green), (b) 594 (yellow), and (c) 633 nm (red). (d) A schematic illustration of the hexagonal photodiode showing the dimensions.

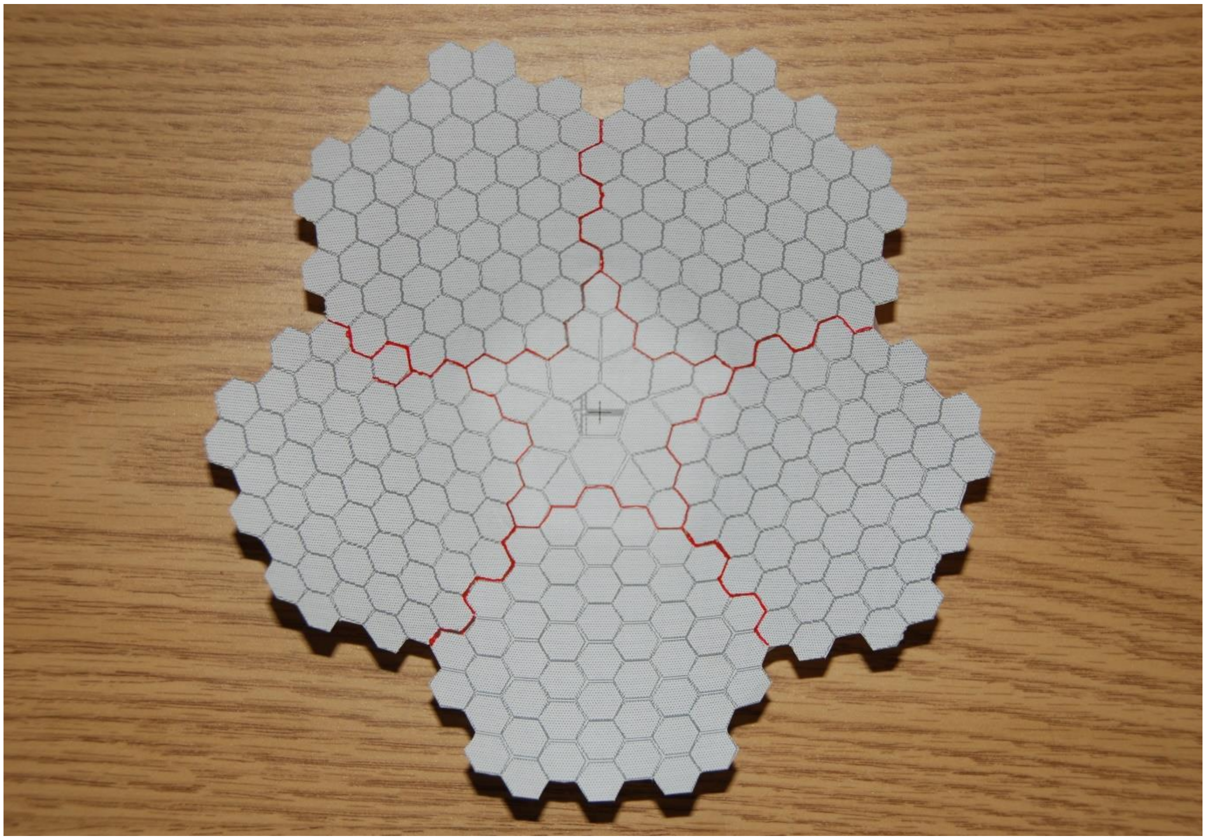

Supplementary Figure 3 | Macroscopic view of the hemisphere formed using subdivided truncated icosahedron represented by paper origami.

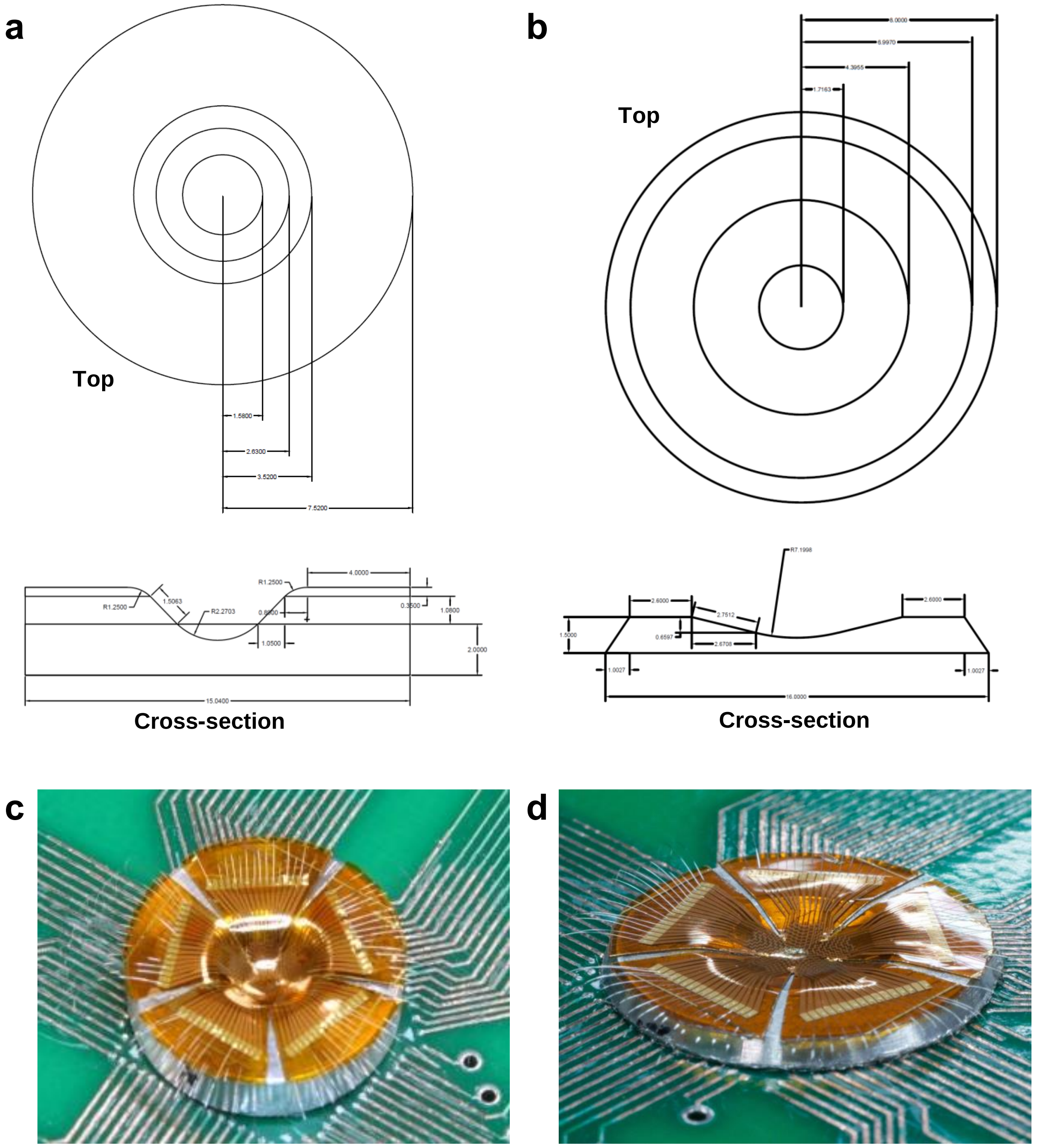

Supplementary Figure 4 | Concave fixtures for mounting a flexible focal plane array (FPA). Fixture dimensions for the FPA with a (a) 2.27 mm radius and (b) 7.20 mm radius. Photographic images of the FPA installed into the fixtures for a (c) 2.27 mm radius and a (d) 7.20 mm radius.

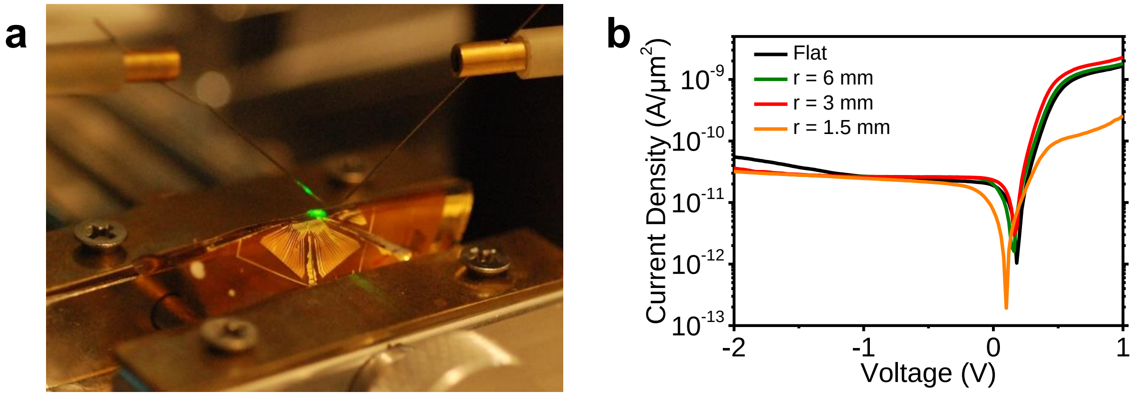

Supplementary Figure 5 | Performance comparison of the flexible photodetector under different bending radii. (a) Photographic image of the flexible silicon photodetector array being analyzed under bending conditions. (b) Current densities of a single photodetector measured under different bending states. Measurements were taken under flat state and bent state with radii of 6 mm, 3 mm, and 1.5 mm. At 1.5 mm radius, the device performance started to degrade.

a

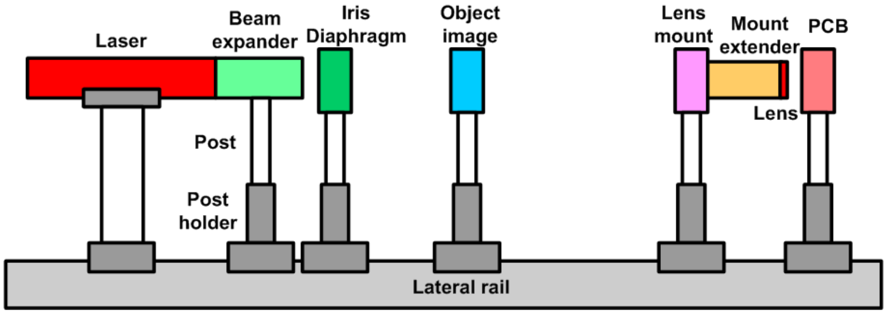

b

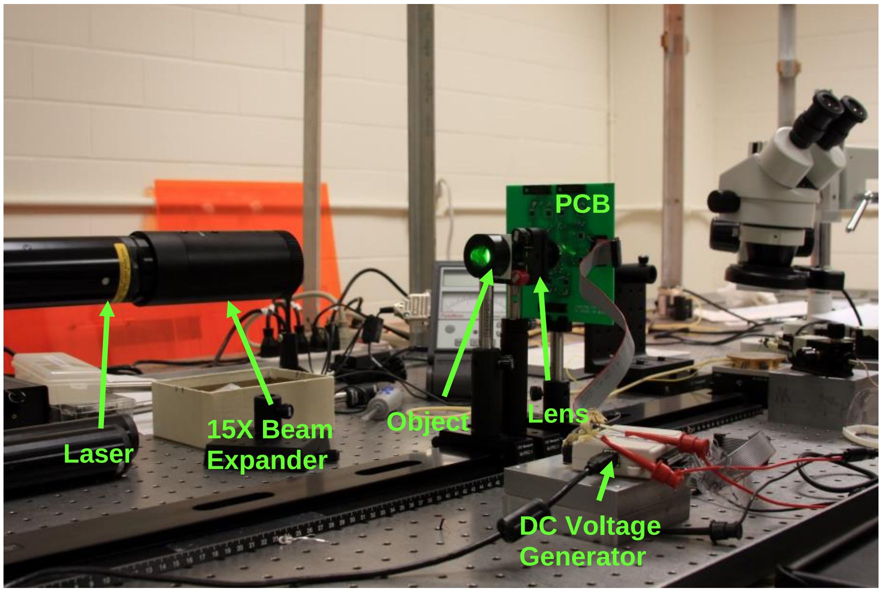

Supplementary Figure 6 | Experimental setup for recording with a concave hemispherical focal plane array. All components were mounted on a lateral rail fixed onto an optics table. (a) Schematic illustration showing the experimental setup. (b) Photographic image of the experimental setup.

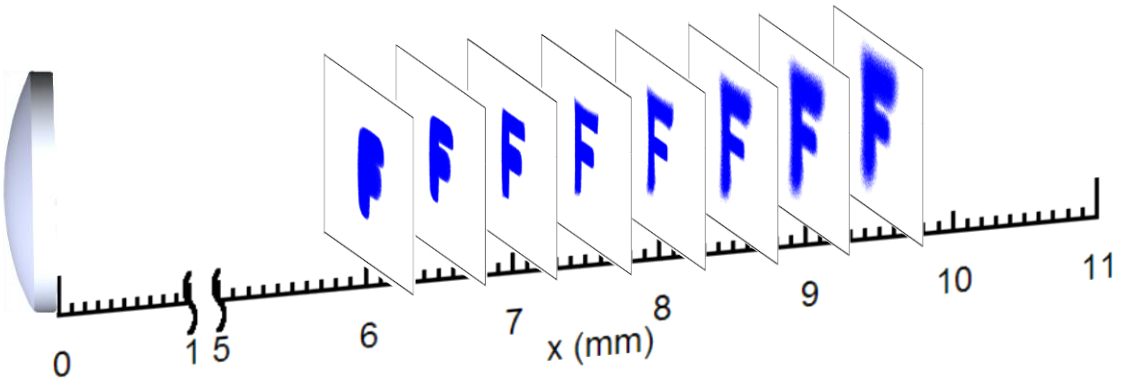

Supplementary Figure 7 | Estimated focal length of the 10 mm diameter plano-convex lens. The estimated back focal length is between 7.0 and 8.5 mm.

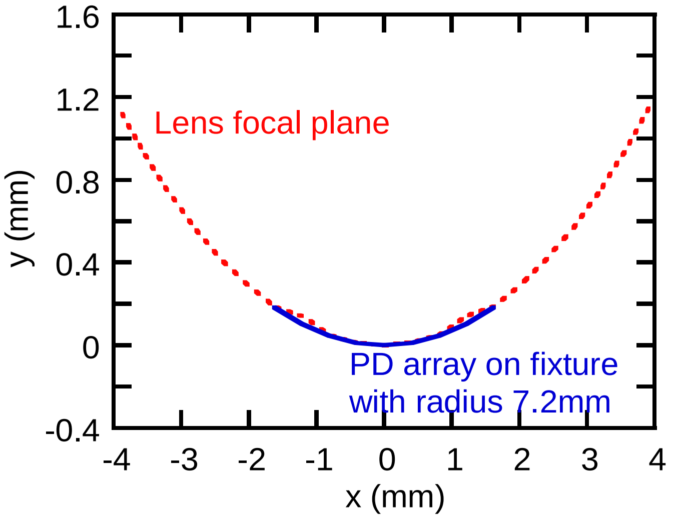

Supplementary Figure 8 | Calculated focal plane of the ray passing through the plano-convex lens (dotted red curve), and the measured focal plane of the silicon optoelectronics array (blue curve) for the concave hemispherical focal plane array with a 7.20 mm radius of curvature. The plano-convex lens used for this experiment had a 10 mm diameter and a 20 mm focal length.

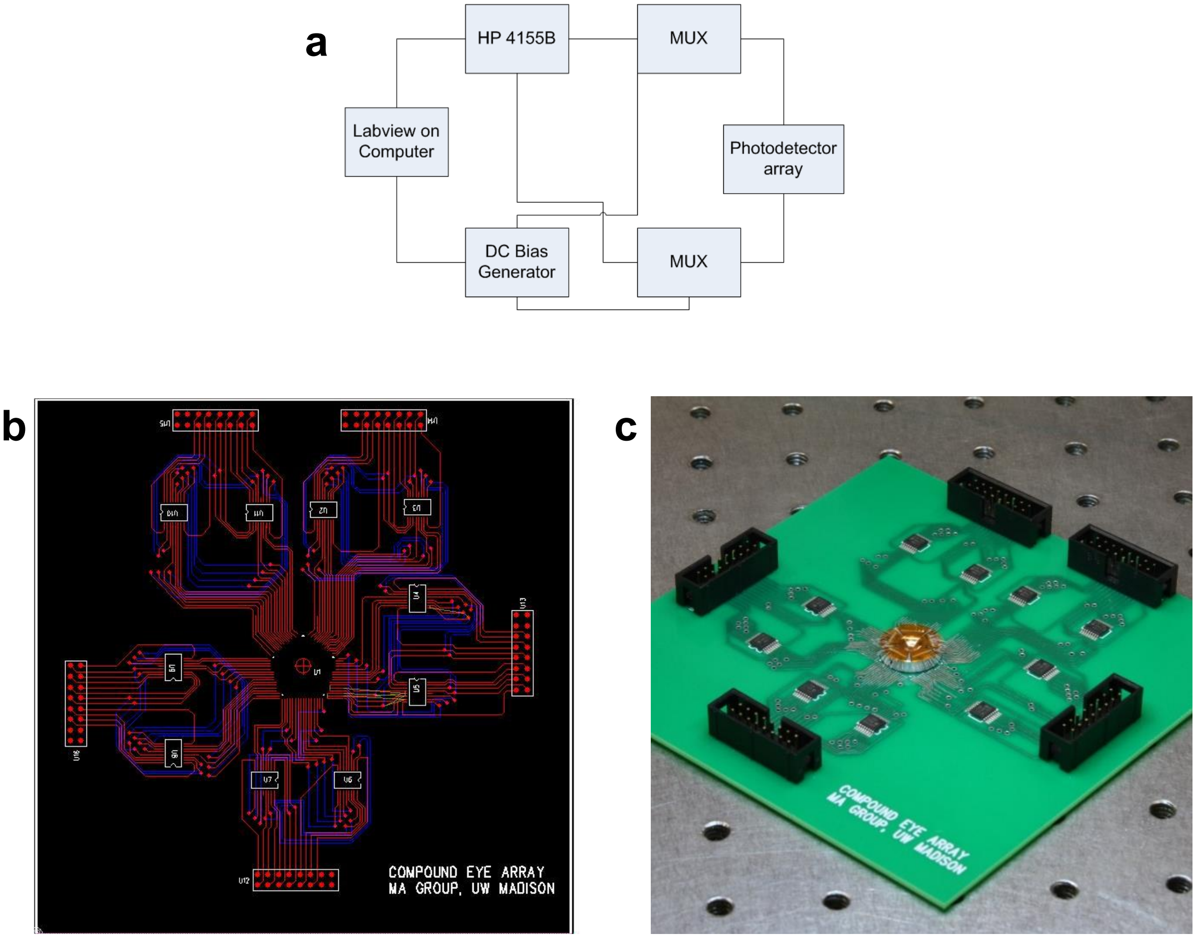

Supplementary Figure 9 | (a) Schematic diagram of the recording mechanism for the hemispherical camera systems used in this study, where multiplexers were used to scan the signals obtained from each photodetector in the array and recorded using LabView coded software. (b) Wiring diagram of the printed circuit board (PCB) where the electronic eyes were mounted onto. Ten low-voltage 4-/8-channel multiplexers (ADG708; Analog Devices) were used in total. (c) Photograph of the concave hemispherical focal plane array mounted on the PCB.

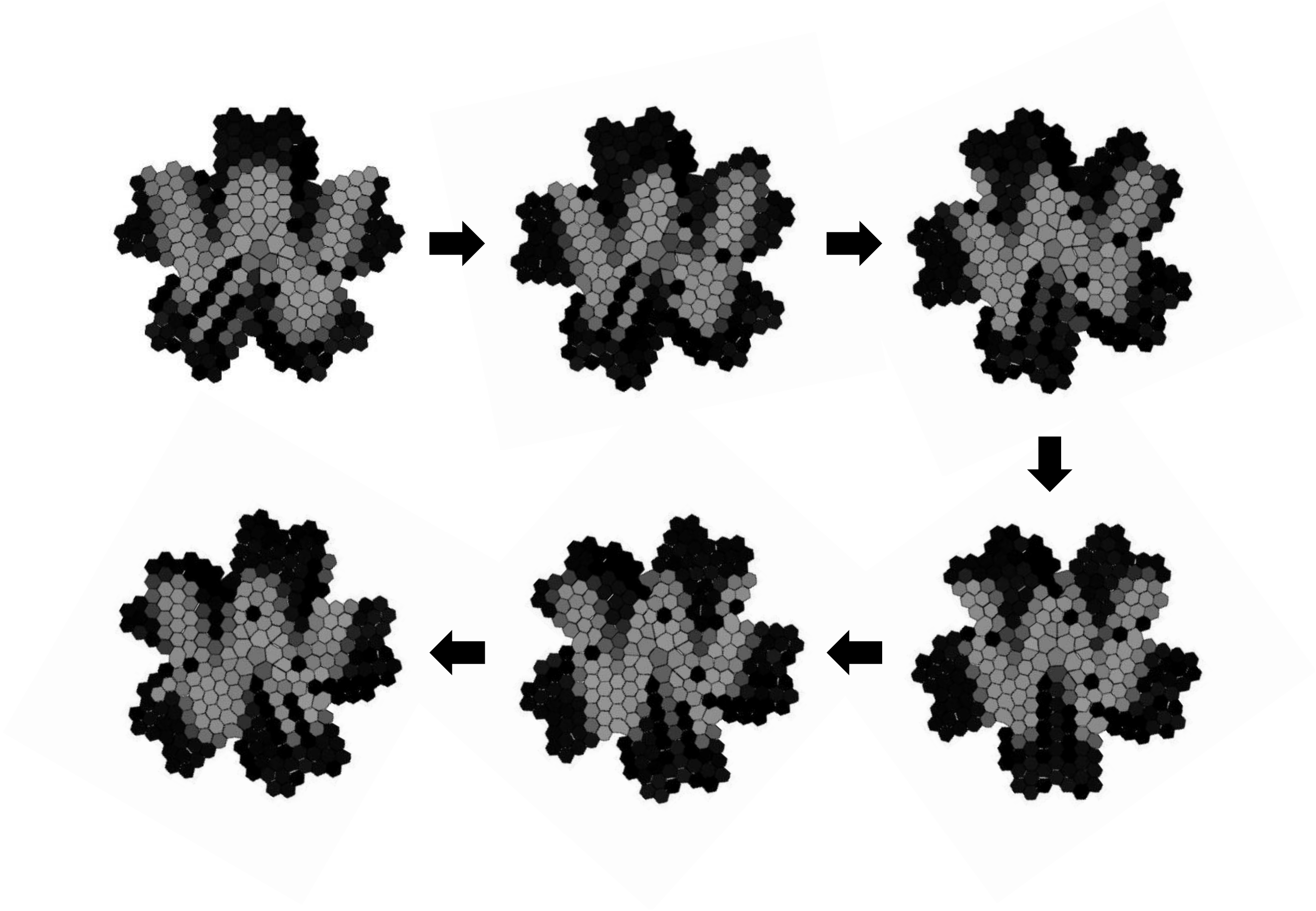

Supplementary Figure 10 | Recorded snapshots of the letter ‘W’ acquired from the hemispherical electronic eye camera with a large radius of curvature (7.20 mm). The image was scanned from 0° to 60° in 12° increments, with the image rotated clockwise.

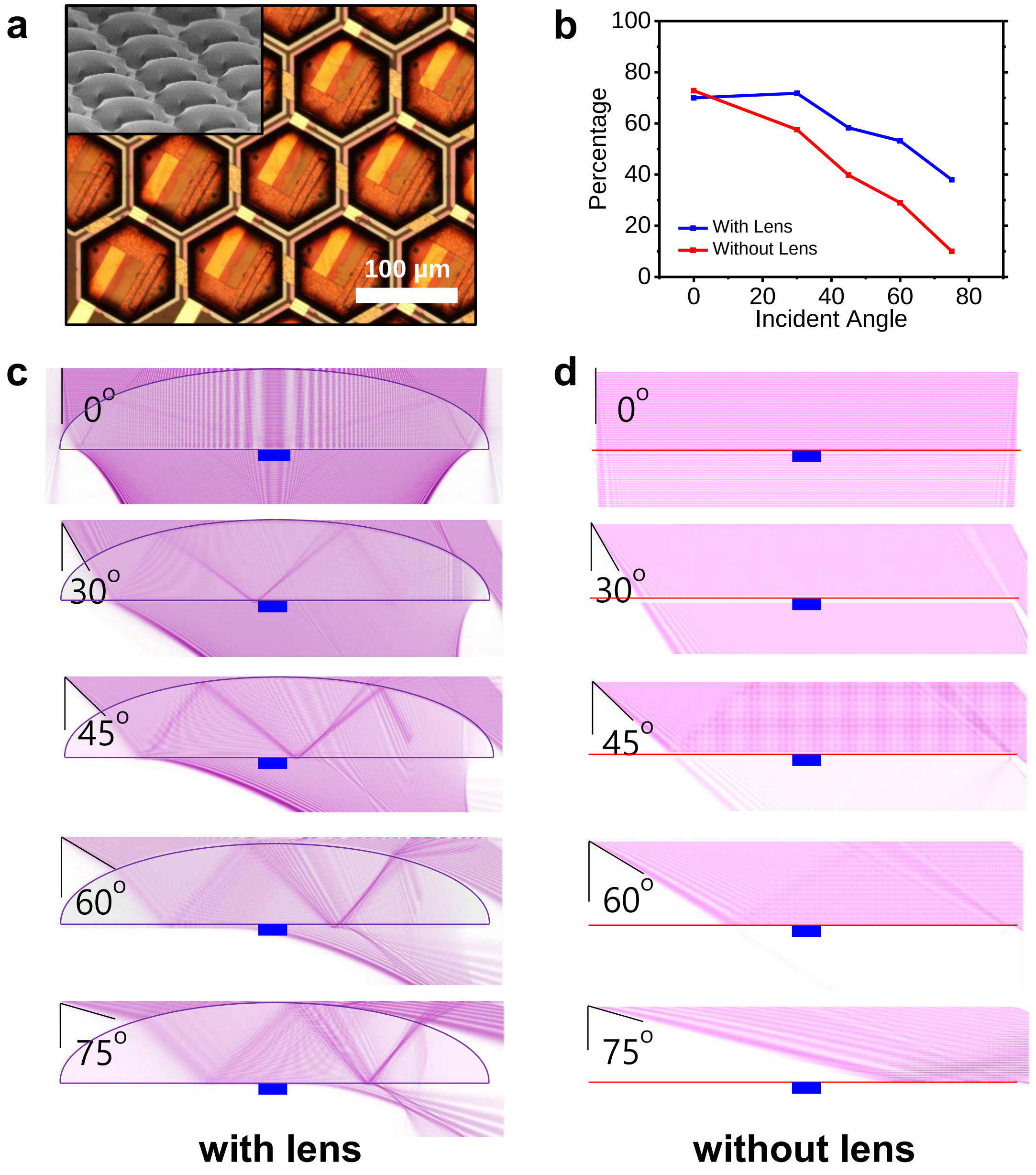

Supplementary Figure 11 | Silicon optoelectronic device properties with polymer microlens integration. (a) Optical microscope image of the silicon photodiode array integrated with polymer microlenses. Inset image shows the scanning electron microscope image of the array. (b) Percentage of light collected by the photodiode with respect to various incident angles with and without a microlens. (c) Ray patterns traced from different incident angles for a single photodetector with a microlens from 0° to 75°. The bold rectangular slab in the center indicates the sensor region of the photodiode. (d) Ray patterns traced from different incident angles for a single photodetector without a microlens from 0° to 75°. The bold rectangular slab in the center indicates the sensor region of the photodiode.

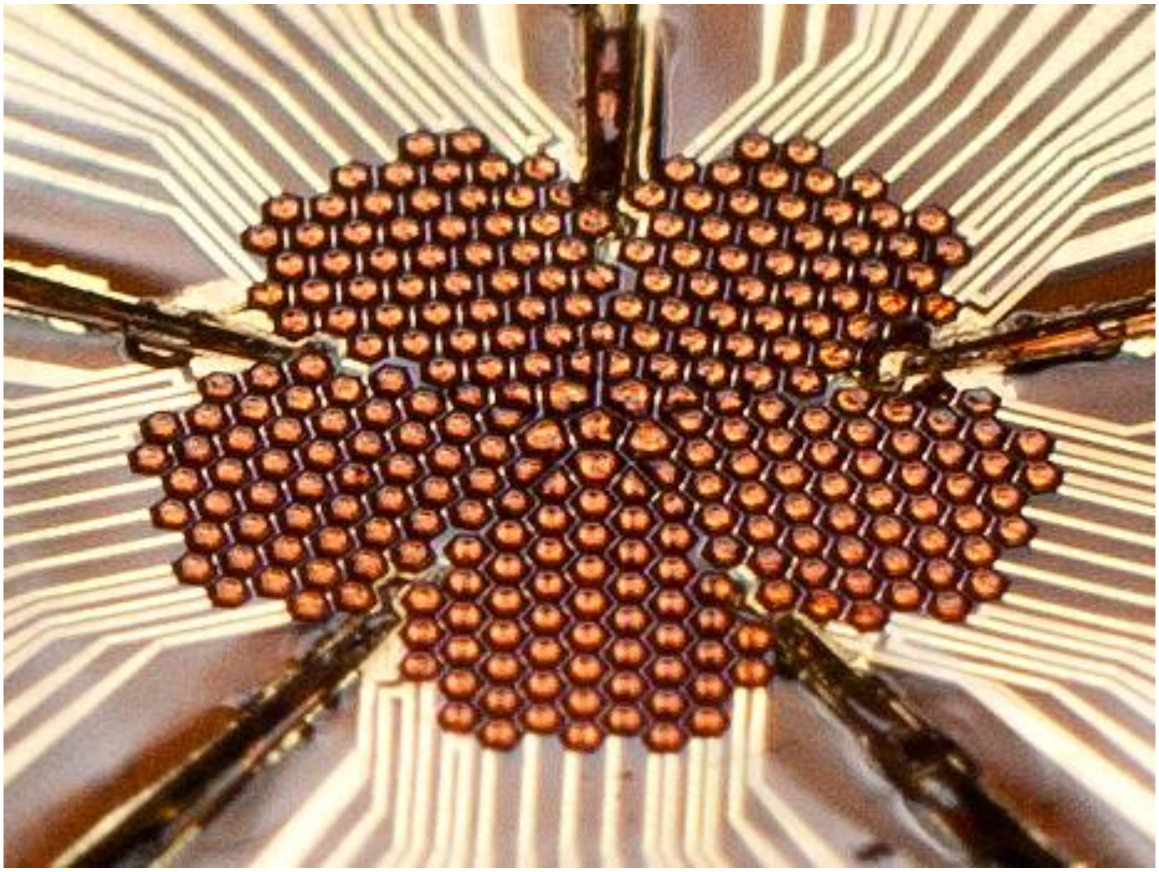

Supplementary Figure 12 | Photographic image of the map of half truncated icosahedron based on a silicon nanomembrane photodetector array with a photoresist microlens on each pixel before folding into a convex hemisphere.

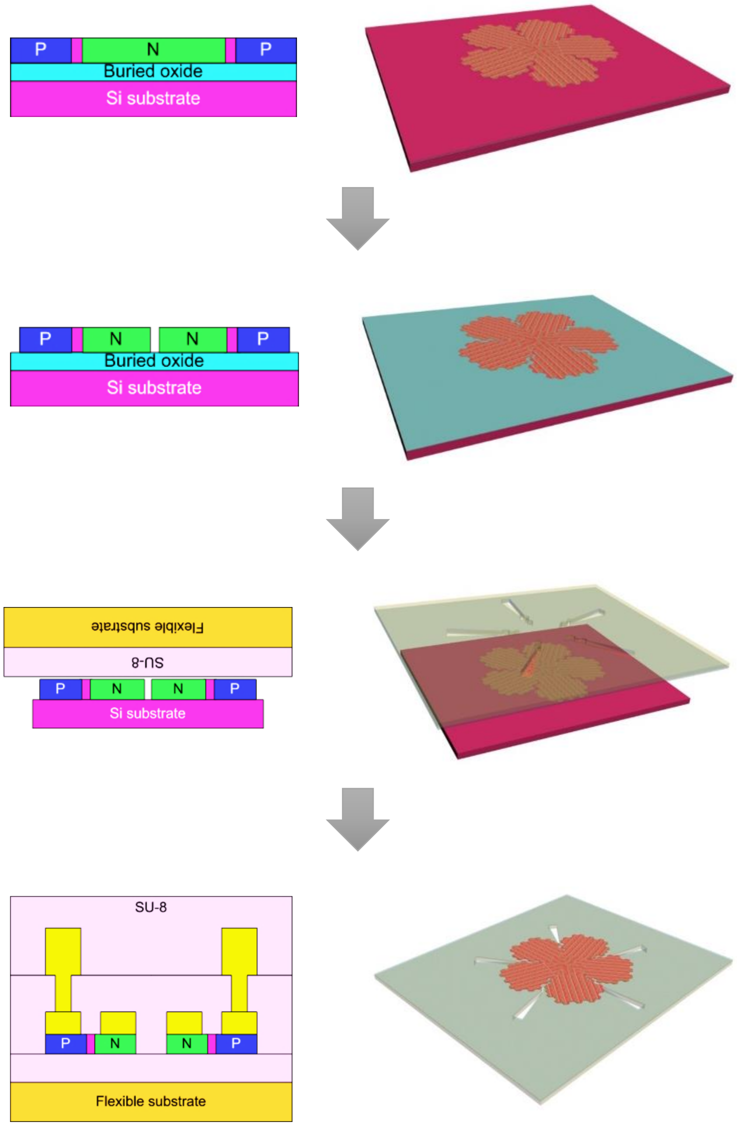

Supplementary Figure 13 | Schematic illustrations showing the detailed fabrication process of the hemispherical electronic eye systems described in the methods section of this report. The two-dimensional schematic illustrations on the left represent cross-section views of the device corresponding to the three-dimensional fabrication steps described on the right.

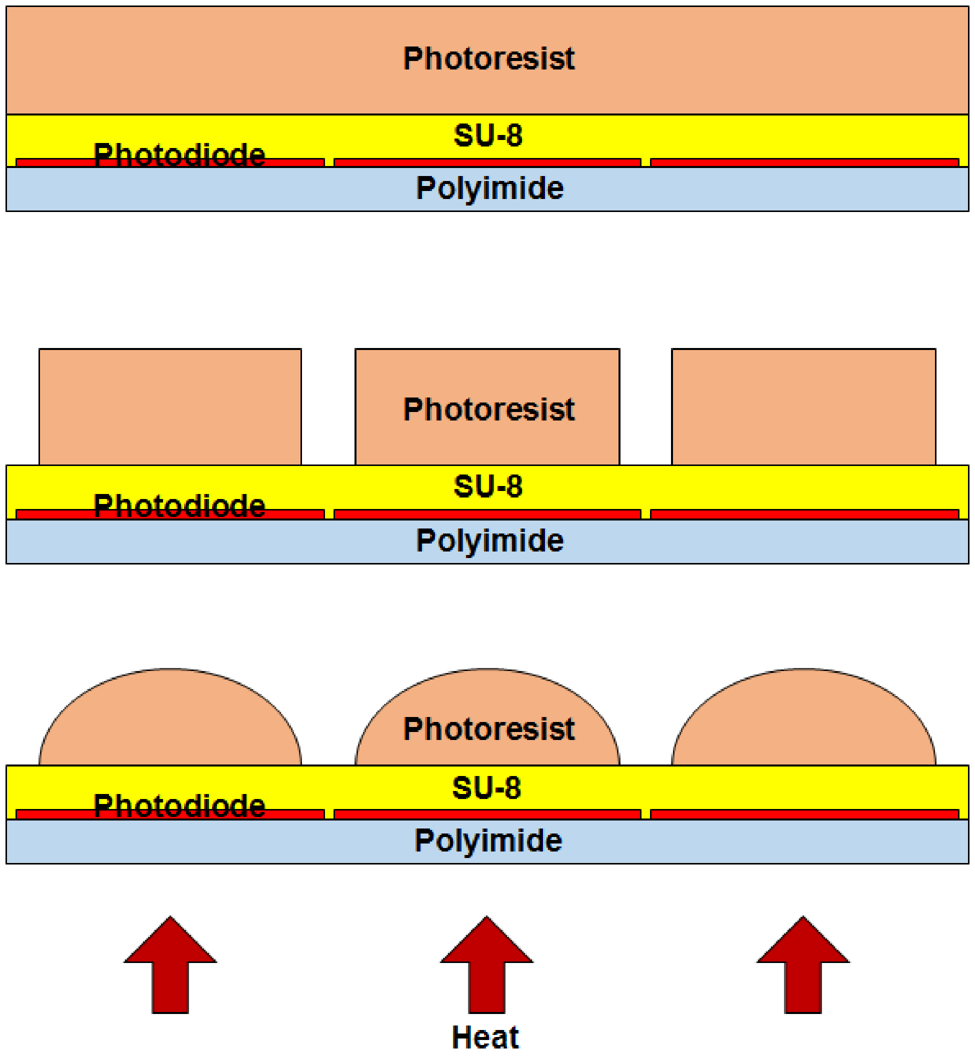

Supplementary Figure 14 | Cross-section schematic illustration showing the photoresist reflow process to create microlenses on top of photodiodes.
